# Detection of Topological Spin Textures via Nonlinear Magnetic Responses

**DOI:** 10.1021/acs.nanolett.1c02723

**Published:** 2021-12-22

**Authors:** Mariia Stepanova, Jan Masell, Erik Lysne, Peggy Schoenherr, Laura Köhler, Michael Paulsen, Alireza Qaiumzadeh, Naoya Kanazawa, Achim Rosch, Yoshinori Tokura, Arne Brataas, Markus Garst, Dennis Meier

**Affiliations:** † Department of Materials Science and Engineering, Norwegian University of Science and Technology (NTNU), Trondheim 7491, Norway; ‡ Center for Quantum Spintronics, Department of Physics, Norwegian University of Science and Technology (NTNU), Trondheim 7491, Norway; § RIKEN Center for Emergent Matter Science (CEMS), Wako 351-0198, Japan; ∥ School of Materials Science and Engineering, University of New South Wales, Sydney, Sydney New South Wales 2052, Australia; ⊥ ARC Centre of Excellence in Future Low-Energy Electronics Technologies (FLEET), UNSW Sydney, Sydney, NSW 2052, Australia; # Institute of Theoretical Solid State Physics, Karlsruhe Institute of Technology, 76049 Karlsruhe, Germany; ∇ Physikalisch-Technische Bundesanstalt (PTB), Berlin 10587, Germany; ○ Department of Applied Physics, University of Tokyo, Tokyo 113-8656, Japan; ◆ Institute for Theoretical Physics, University of Cologne, Cologne 50937, Germany; ¶ Tokyo College, University of Tokyo, Tokyo 113−8656, Japan; ■ Institute for Quantum Materials and Technology, Karlsruhe Institute of Technology, 76021 Karlsruhe, Germany

**Keywords:** chiral magnets, domain walls, FeGe, magnetic force microscopy, nonlinear magnetic response, spintronics, topological order

## Abstract

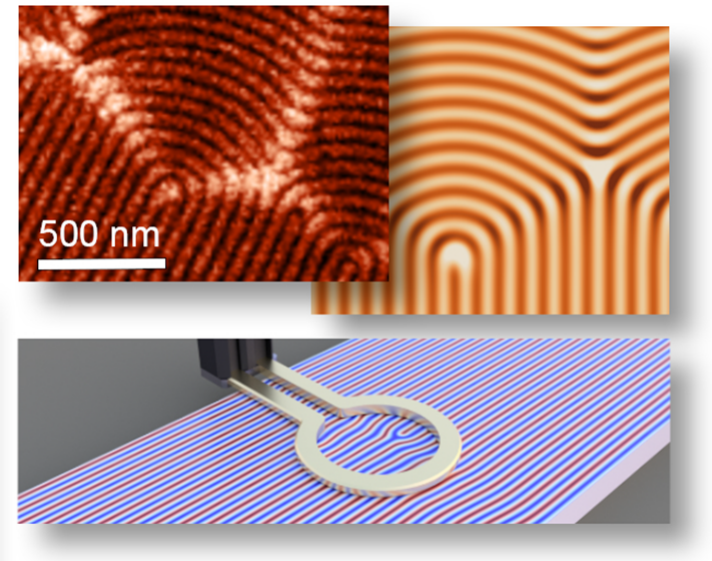

Topologically nontrivial spin textures, such as skyrmions and dislocations, display emergent electrodynamics and can be moved by spin currents over macroscopic distances. These unique properties and their nanoscale size make them excellent candidates for the development of next-generation race-track memory and unconventional computing. A major challenge for these applications and the investigation of nanoscale magnetic structures in general is the realization of suitable detection schemes. We study magnetic disclinations, dislocations, and domain walls in FeGe and reveal pronounced responses that distinguish them from the helimagnetic background. A combination of magnetic force microscopy (MFM) and micromagnetic simulations links the response to the local magnetic susceptibility, that is, characteristic changes in the spin texture driven by the MFM tip. On the basis of the findings, which we explain using nonlinear response theory, we propose a read-out scheme using superconducting microcoils, presenting an innovative approach for detecting topological spin textures and domain walls in device-relevant geometries.

The discovery of magnetic skyrmions^1−4^ and their emergent physical properties^5−12^ propelled the research on topological spin states in solid state systems and motivated new concepts for spintronics devices where skyrmions act as mobile information carriers.^13−16^ Skyrmions are intriguing as they are nanoscale objects that efficiently couple to spin currents, enabling high storage density and low-energy control.^4,6,17^ With the progress of the field, the scope widened and other spin textures, such as merons,^18−20^ biskyrmions,^21^ and hopfions^22,23^ have been considered. Recently, disclinations, dislocations, and helimagnetic domain walls emerged as a new family of topological nanosystems that naturally arise in the helimagnetic ground state in chiral magnets.^24−27^ The emergence of these topological spin textures is enabled by the lamellar-like morphology of the helimagnetic order analogous to, for example, cholesteric liquid crystals,^28^ swimming bacteria,^29^ and the skin on our palms.^30^ In magnetism, certain analogies exist to ferromagnetic stripe domains,^31^ but the involved length scales are substantially different. In chiral magnets, the spin structure twists continuously and the periodicity is up to 3 orders of magnitude smaller than for the classical stripe domains.^25^ Edge dislocations within the helimagnetic structure are formed by a pair of +π and −π disclinations and, depending on their Burgers vector, can carry a topological charge . Such dislocations are topologically equivalent to half-skyrmions or merons as discussed in ref (26). Both disclinations and edge dislocations arise even without the external magnetic field usually needed to stabilize skyrmions and represent important building blocks for the formation of helimagnetic domain walls. It is now established that spin textures with nontrivial topology hold great technological potential enabling, for example, reconfigurable logic gates,^15,32^ race track memory,^13,14^ as well as neuromorphic^33^ and reservoir computing.^34^ Sensing of individual topological spin structures and magnetic nano-objects in general, in a way that is compatible with the proposed device architectures and semiconductor fabrication methods, however, remains a challenging task. Topological spin arrangements have been resolved by various imaging techniques, including electron,^13,35^ X-ray,^9,36^ and magneto-optical^9^ microscopy, as well as scanning tunneling microscopy,^36,37^ magnetic force microscopy (MFM),^38,39^ and nitrogen vacancy magnetometry.^40^ While these methods have provided important insight into the physics of topologically nontrivial spin textures, they are not directly transferrable to devices. For the specific case of skyrmion-electronics, a promising method is to utilize the topological Hall effect,^41,42^ or magnetoresistance measurements,^43^ but an expansion toward other magnetic nanoentities remains to be demonstrated. Thus, the development of dynamical and more agile read-out schemes that allow for resolving individual nanoscale spin textures in device-relevant geometries is highly desirable.

Here, we demonstrate how nonlinear magnetic responses can be utilized to detect and identify both topologically trivial and nontrivial spin textures at the nanoscale. Combining MFM and micromagnetic simulations, we analyze the local magnetic response of magnetic disclinations, dislocations, and helimagnetic domain walls in the model system FeGe. Our results clarify the local magnetic properties and reveal characteristic fingerprints that enable selective detection of different nanoscale spin arrangements using superconducting microcoils. The general feasibility and universality of this approach are demonstrated by two examples, considering the signal formation for an edge dislocation () as well as a topologically trivial curvature domain wall (*W* = 0).

FeGe belongs to the family of helimagnets with B20 structure^3,4,44−46^ and its magnetic phase diagram is well-established.^47^ In addition, diverse nanoscale spin textures have been observed, and their equilibrium structure has been analyzed in detail, rendering FeGe an ideal model system for this work. FeGe develops helimagnetic order below *T*_N_ = 280 K, stabilized by the competition between Heisenberg exchange and the relativistic Dzyaloshinskii-Moriya interactions in the noncentrosymmetric crystal lattice. The helimagnetic ground state is characterized by a gradual rotation of the magnetization vector **M** about a wave vector , where λ = 70 nm^48^ is the helical period and **q̂** characterizes the direction of the helical axis (Figure 1a).

**Figure 1:**
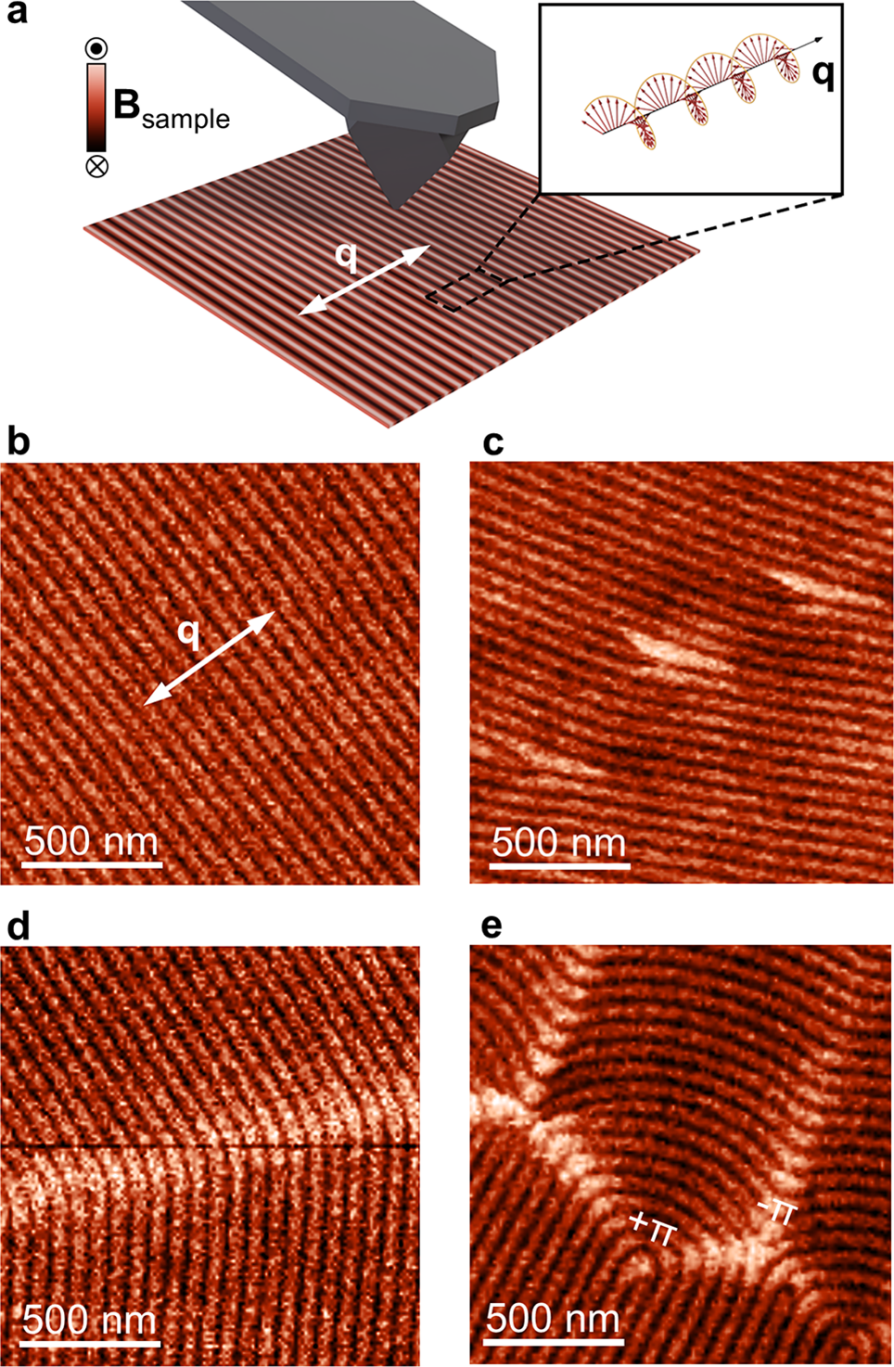
MFM imaging of helimagnetic order, dislocations, and domain walls. (a) Schematic illustration of the helical spin structure in FeGe described by the wave vector **q** and the characteristic stripelike pattern probed by MFM in the helical phase. The color scale indicates the direction of the magnetic stray field **B** from the sample. (b) MFM image of the helimagnetic order within a single **q** domain in FeGe. Note that the MFM contrast originates from the spin helix, giving rise to a lamellar morphology with a measured periodicity of ∼70 nm, which is about 3 orders of magnitude smaller than the conventional stripe domains in ferromagnetic systems. In FeGe, domains are formed only on much larger length scales as seen, for example, in (d,e), corresponding to regions with a different orientation of **q**. (c–e) MFM images showing magnetic dislocations in the lamellar-like spin structure (c), a curvature domain wall (d), and a zigzag domain wall composed of +π and −π disclinations (e). All 1D and 2D spin textures in (c–e) exhibit enhanced bright MFM contrast compared to the helimagnetic background.

Figure 1b shows an MFM image of FeGe in the helimagnetic phase, recorded in two-pass mode (see Supporting Information Note 1 for details). Bright and dark lines reflect the periodic magnetic structure with λ ≈ 70 nm and **q** lying in the surface plane (white arrow) in agreement with neutron scattering data.^48^ Because of the lamellar morphology,^25,26^ which is analogous to cholesteric liquid crystals, different types of defects arise in FeGe at the nanoscale including topologically nontrivial objects such as dislocations (Figure 1c) and zigzag disclination walls (Figure 1e) as well as more simple curvature walls that do not carry a topological charge (Figure 1d). Details about the inner structure of the different spin textures are reported elsewhere.^25,26^ Most importantly for this study, Figure 1c–e reveals a universal feature that is shared by all defect structures, independent of their topology, shape, and dimensionality. All defects exhibit an additionally enhanced contrast in the MFM data that is not observed in regions with perfect lamellar-like order (Figure 1b), separating them from the helimagnetic background. A similar MFM response has been observed at the helimagnetic domain walls in earlier studies but without clarifying the microscopic origin.^26^ Thorough examination of the data in Figure 1 reveals that the enhanced MFM response is asymmetric: only the bright lines, which indicate an attractive force between probe tip and sample, exhibit increased intensity and width. Furthermore, we find that the enhanced MFM signal can be detected more than 100 nm above the surface, that is, before the actual spin structure of the defects is resolved (see Figure S1 and Figure S2).

In order to understand the unusual local response of the magnetic defects, we conduct micromagnetic simulations. The MFM signal is proportional to the phase shift *Δϕ* ∝ −d^2^*E*_int_/d*z*_0_^2^ of the oscillating probe tip. *E*_int_ is the dipolar interaction energy between the tip and sample and *z*_0_ denotes the tip–sample distance. First, we theoretically discuss *E*_int_ on the level of linear response. In the linear-response limit, the probe picks up the magnetic signal of the unperturbed system, that is, its coupling to the sample and influence on the magnetization is neglected, leading to
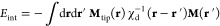
1Here, **M**_tip_ is the magnetization of the MFM tip, **M** is the magnetization in the sample, and χ_d_^–1^ is the dipolar interaction between the tip and the sample (see Supporting Information Note 5). **M**_tip_ is often approximated by a point-dipole (**M**_tip_(**r**) = **m**_tip_δ^3^(**r**–**r**_0_)); **m**_tip_ is the magnetic moment of the tip and **r**_0_ its position). The stray field **B** of the helical phase is shown in Figure 2a. It decreases exponentially as a function of the distance,^25^ with λ/2π determining the decay length of the stray field, see Supporting Information Note 5. Microscopically, **B** is generated by magnetic bulk charges, ∇·**M**, and surface charges, **n**·**M** (**n** is the unit vector normal to the surface). As bulk charges are absent in the case of an ideal spin helix, **B** is dominated by surface charges, as shown in Figure 2a,c. Thus, taking into account that the projected periodicity λ_p_ of the lamella-like order increases whenever the helimagnetic structure is bent, that is, λ_p_ > λ, the generally stronger magnetic stray field **B** and phase shift *Δϕ* at defects can be explained based on an increased decay length  associated with local magnetic surface charges (see Figure S.3, S.4, Supporting Information Note 5). However, while this geometrically driven effect can give rise to higher attractive and repulsive forces at defect structures, it is qualitatively different from the asymmetric effect presented in Figure 1, failing to explain why only attractive forces appear amplified at the defect sites.

**Figure 2:**
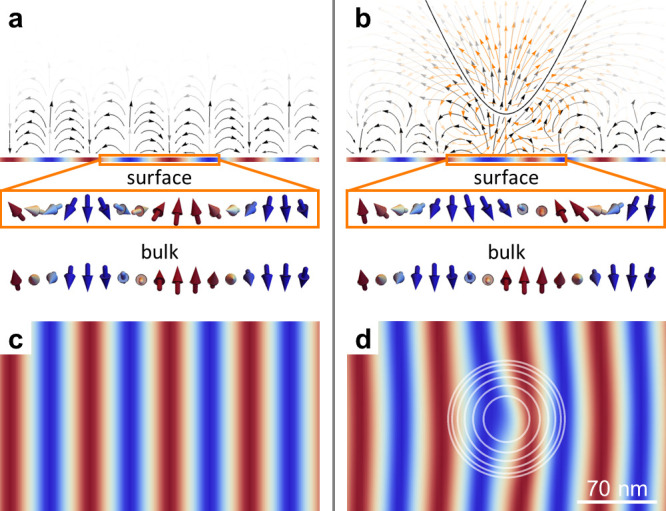
Calculated local response of the helimagnetic spin structure. (a) Side view of the helimagnetic order which–in the absence of an invasive magnetic tip–differs only slightly for the surface (top) and the bulk (bottom). The out-of-plane magnetic components associated with the helical spin structure (sketched by solid black arrows) generate alternating magnetic surface charges (blue to red) with a periodicity of about 70 nm, which are the main source for the magnetic stray field (curved black arrows, saturation encodes field strength). (b) Same as in (a) in the presence of an invasive magnetic tip (**m**_tip_ = 10^–16^ A m^2^, positioned at distance *z*_0_ = 40 nm from the surface), leading to substantial changes in the helimagnetic structure at the surface (top) compared to the bulk (bottom). The extra field of the tip in (b), approximated by a single dipole, is colored orange. (c) Top view of the alternating magnetic surface charges seen in (a). (d) Same as in (c) in the presence of an invasive magnetic tip. The position of the MFM tip is indicated in (d) by white rings, each corresponding to a factor of 2 decreased magnetic field. The polarizing influence of the tip in (b,d) is clearly visible.

This discrepancy leads us to the conclusion that the dipolar interaction between the magnetic moment of the tip and the stray field of the spin helix is non-negligible.^31^ Therefore, we go beyond the linear response theory that describes noninvasive MFM measurements and include the local stray field of the magnetic MFM tip in our three-dimensional simulations. This approach becomes important in cases where the probe couples more strongly to the system so that it directly influences the response and the linear approximation breaks down. For mesoscopic magnetic domains, this effect has been observed, for example, in garnet thin films.^31^ To account for such emergent nonlinear responses in the helimagnetic spin texture of FeGe, we model the tip by a single dipole moment and the magnetization is described by a lowest order gradient expansion
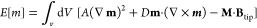
2subject to the boundary conditions of the embedding helical phase in three spatial dimensions (**m** = **M**/**M**_s_ is the normalized magnetization, **M**_s_ is the saturation magnetization, *A* is the exchange stiffness, *D* is the Dzyaloshinskii-Moriya interaction, and **B**_tip_ is the dipolar stray field that is emitted by the tip). For simplicity, we neglect effects from the demagnetizing field and cubic anisotropies as they do not change the results qualitatively (see Supporting Information Note 1 for details on parameters and software). The calculated helimagnetic texture resulting under realistic experimental conditions is presented in Figure 2b,d. As function of distance *z*_0_, the stray field from the tip decays as **B**_tip_ ∝ *z*_0_^–3^ in the dipole approximation; see Supporting Information Note 5 for a discussion of pyramidally shaped tips. As a consequence, the strongest polarizing effects in the spin helix are observed at the sample surface, where the tip induces an additional magnetic surface charge (blue in Figure 2d). Vice versa, we find that the net-induced surface charge is the main source for the magnetic stray field probed by the tip (bent black lines in Figure 2b), leading to a stronger and more long-ranged attractive force (polynomially decaying instead of exponentially) compared to the unperturbed helimagnetic structure displayed in Figure 2a. Note that this net polarization of the magnetization occurs for helimagnetic order both with and without defect structures (see Supporting Information Note 5 for details). In summary, the magnetization of the tip leads to an additional nonlinear response in MFM. In a second order process, **M**_tip_ creates magnetic charges within the sample via dipolar interactions which then feed back onto the tip
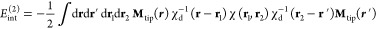
3

The efficiency for inducing magnetic charges, however, is set by the magnetic susceptibility χ, so that local variations in χ can lead to additional contributions in MFM. At the level of domains, such additional contributions have been studied intensively and are known as susceptibility contrast.^31^ Our calculations reveal that such susceptibility contributions are equally important at the nanoscale, leading to substantially different nonlinear responses for spin-helix segments with different out-of-plane magnetization components.

To verify that the local susceptibility contrast observed at helimagnetic defects originates from tip-induced magnetic surface charges, that is, a nonlinear response, we simulate MFM scans with oppositely magnetized tips. As an instructive example, we consider the case of a topologically nontrivial zigzag wall containing +π and −π disclinations.^26^ For reference, the magnetic surface charges of the ideal, undisturbed zigzag domain wall are presented in Figure 3a. Simulations accounting for the tip–sample interaction are presented in Figure 3b,d. The simulations show that the enhanced magnetization of the +π and −π disclination centers inverts as the magnetization of the tip is switched from “down” (Figure 3b) to “up” (Figure 3d). In contrast, the lines connecting the +π and −π disclinations remain bright upon reversal, corresponding to an attractive force on the tip independent of the orientation of **M**_tip_(***r***). Corresponding experimental MFM scans on a real sample with down and up magnetized tip are presented in Figure 3c,e, respectively (see Supporting Information Note 1 for experimental details). Both MFM images in Figure 3c,e reveal qualitatively the same spin texture at the zigzag wall. In agreement with the simulations, we observe that the MFM signal at the disclination center inverts (marked by white dashed circles). The signal associated with the domain wall, however, remains bright in both scans (marked by white dashed lines to the right of the circles). The data thus confirms an attractive force that occurs at the site of the domain wall due to tip-induced magnetic surface charges in the helical spin structure, leading to pronounced susceptibility contrast in MFM.^31,49−51^ As the magnetization at defects deviates from the energetically favorable helical structure, it is reasonable to assume that it is more susceptible to external magnetic fields. This assumption is corroborated by our numerical calculations of the susceptibility χ in Supporting Information Note 6. The higher susceptibility leads to a more efficient generation of surface charges than in the helimagnetic background and, hence, an additional long-ranged attractive force, consistent with our MFM measurements (Figure S.1, S.2, S.5–S.8). In contrast to the MFM contributions from magnetic surface charges, however, the local susceptibility contrast is proportional to ***m***_tip_^2^ (Supporting Information Note 5). As a consequence, susceptibility-related signals linked to the zigzag wall do not invert along with the tip magnetization and can be isolated by adding MFM images gained with opposite tip magnetization. The latter is presented in Figure S.9–S.11 where the MFM sum image reveals a pronounced contrast associated with the curved helix structure, confirming that defects in the helimagnetic structure in FeGe exhibit a locally enhanced susceptibility. We note, however, that the spin structures recorded with opposite tip magnetization are not identical as the experiment required heating of the sample above *T*_N_ to invert the tip magnetization. Thus, a reliable isolation of susceptibility contributions is possible only in specific regions as presented in Figure S.10, corresponding to the area marked by the dashed white line in the larger scan in Figure S.11.

**Figure 3:**
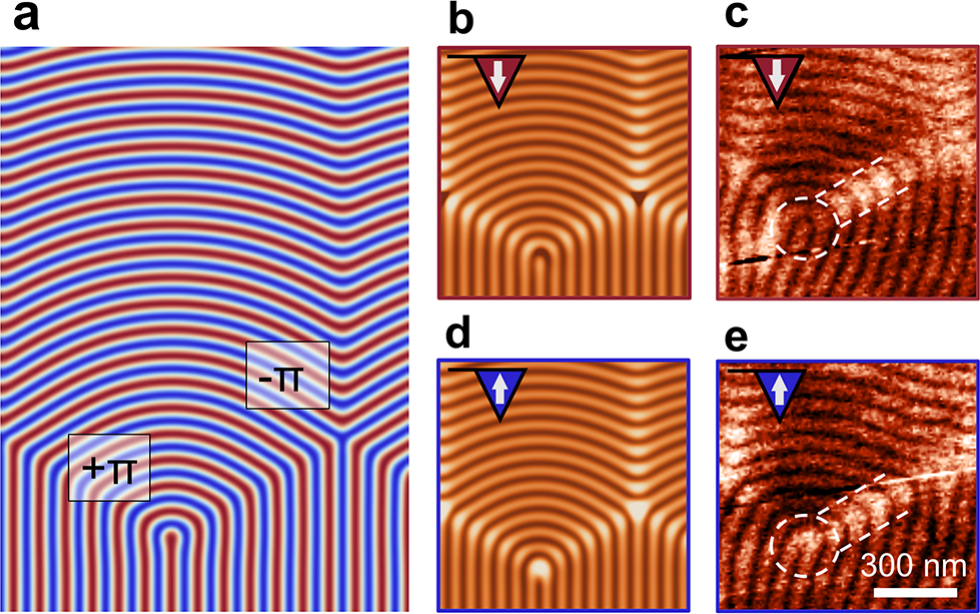
Magnetic response from a zigzag domain wall with alternating +π and −π disclinations as a function of the orientation of the tip magnetization. (a) Calculated magnetic surface charges of a zigzag domain wall at the surface of FeGe. The color denotes the out-of-plane magnetization related to the spin helix from pointing up (blue) to down (red). (b,d) Calculated nonlinear MFM response for a down (red, b) and up (blue, d) magnetized tip, taking the tip–sample interaction into account (lift height, 100 nm; tip moment, 2 × 10^–16^ A m^2^). Bright and dark colors indicate attractive and repulsive forces, respectively. The pattern of bright and dark lines associated with the spin helix inverts as the tip changes magnetization direction, whereas an additional attractive force is detected at the domain wall position independent of the tip magnetization. (c,e) Corresponding MFM images of a zigzag domain wall recorded at the same position with (c) tip magnetized down and (e) up. The size of the scanning area is 1 μm × 1 μm. The white dashed circles mark the center of the disclination, and the white dashed lines mark the domain wall.

In summary, we have identified a pronounced nonlinear response at defects of helimagnetic order in FeGe due to their specific surface polarization induced by the magnetic tip (Figure 2). This nonlinear response occurs as a magnetic field is applied, providing an additional opportunity for sensing and distinguishing nanoscale spin textures. In Figure 4, the latter is shown for selected examples of 1D- and 2D-defects; that is, a magnetic edge dislocation (topologically equivalent to a half-skyrmion or meron) and a topologically trivial curvature wall. The basic detection scheme is presented in Figure 4a, showing an artistic view of a racetrack-like geometry where a stationary superconducting quantum interference device (SQUID) coil detects passing defects. To allow for an insulating spacer layer that separates the read-out coil from the track, we assume the distance between coil and track to be 10 nm. A realistic diameter for the SQUID coil is *r* = 500 nm, which is comparable to state-of-the-art SQUID-on-tip technology.^52,53^ Such nanoSQUIDs can be operated both without and with magnetic background fields, facilitating a sensitivity of 0.6 μ_B_ Hz^–1/2^ at 1 T as demonstrated in ref (54). The local magnetic field required to measure nonlinear responses is generated by a magnet situated below the track. One possibility to generate magnetic field is to follow the established SQUID-on-tip design, fabricating a superconducting microcoil with a nonmagnetic quartz core to support the desired geometry and ensure stability.^52^ Considering the rapid and ongoing progress in the field, the application of patterned nanomagnets as explored in ref (55) as well as single- or multicoil magnets represent additional promising future pathways. Assuming a single-coil magnet with the same diameter as the read-out coil and a cylindrical shape with a 50 μm × 100 nm cross section, a magnetic field of 100 mT can be achieved at a current density of approximately 8 × 10^5^ A cm^–2^. This requirement is compatible with the critical current of Nb coils, which is in the order of several MA/cm^2^ (see, e.g., ref (56)). Thus, using SQUIDs it is feasible to measure both linear and nonlinear responses, detectable as a variation in the magnetic flux ΔΦ. To quantify and compare the expected signals, we assume an idealized geometry where a magnetic field of 100 mT is produced by the same coil as used for detection (Figure 4b–e).

**Figure 4:**
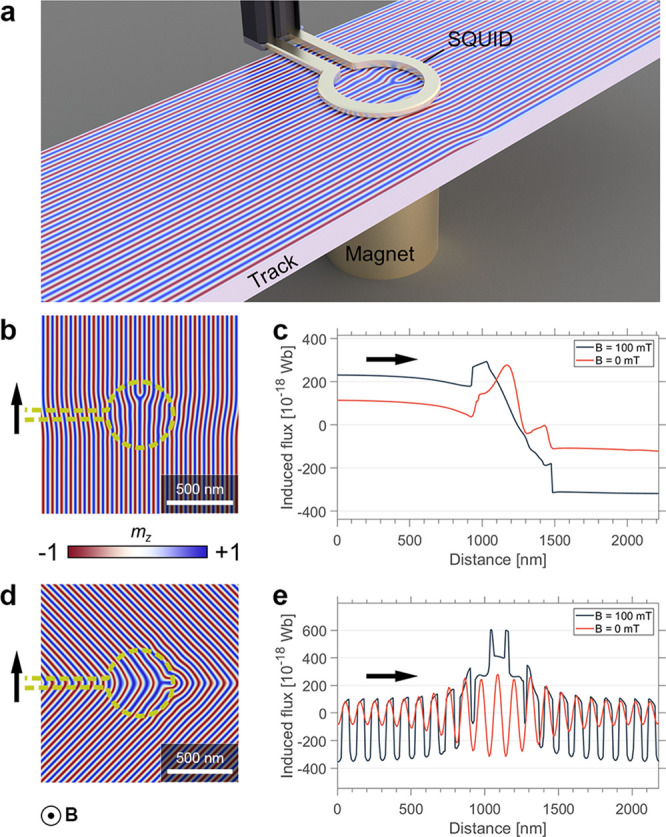
SQUID-based read-out scheme for the detection of 1D and 2D magnetic spin textures. (a) Schematic illustration of a magnetic track with a SQUID coil for read-out (diameter: 500 nm) and a magnet that provides the local field for nonlinear response measurements, presenting the basic setup for detection. Using stationary coils, mobile topological spin textures, here a dislocation, can be sensed and counted via a defect-specific change of the magnetic flux through the coil. (b) Out-of-plane magnetization, **m**_*z*_, of an edge dislocation under the influence of the magnetic stray field (100 mT) from the coil illustrated by the yellow dashed line. **B** gives the direction of the magnetic field within the coil and the black arrow indicates the direction of motion relative to the edge dislocation. (c) Induced magnetic flux measured with biased (100 mT) and nonbiased (0 mT) coils. (d,e), Same as in (b) and (c) for a curvature wall.

The calculations in Figure 4b,c show that for a magnetic dislocation, which binds a finite magnetic surface charge, both linear (0 mT) and nonlinear (100 mT) detection is possible, yielding comparable changes in the magnetic flux. The 180° phase jump, which is induced as dislocations move through the helimagnetic background,^25^ however, is more pronounced when applying nonlinear detection. In contrast to edge dislocations, curvature domain walls exhibit alternating surface charges that macroscopically average to zero, so that the linear signal can effectively cancel out. However, these defects are then still detectable via the nonlinear interaction (Figure 4d,e) which generates a clear peak at the domain wall. This peak makes the signal asymmetric so that it remains detectable even when its oscillating fine-structure cannot be resolved. On the basis of the calculations, a signal span in magnetic flux of ΔΦ ∼ 10^–16^ Wb ≈ 0.2 Φ_0_ is expected. For the coil in Figure 4, this difference translates into a magnetic field ΔΦ/πr^2^ ≈ 510 μT, which is readily measurable using SQUID magnetometers. Importantly, the variation in magnetic flux presented in Figure 4c,e is specific to the cases depicted in Figure 4b,d; in general, the measured signal depends on the coil geometry and position, as well as the direction of movement of the spin texture relative to the coil, providing additional information about the magnetic order at the nanoscale. For example, the magnetic flux is constant for translations perpendicular to the **q**-vector of the spin helix but varies in the direction parallel to **q**, allowing to resolve phase jumps (Figure 4c) and spatial modulations (Figure 4e) in the helimagnetic spin structure.

On the one hand, the nonlinear detection scheme is compatible with local imaging techniques such as scanning SQUID microscopy,^57^ where the coil is scanned across the sample surface to detect the defects, removing the requirement of sub-100 nm resolution to verify emergent defect structures. On the other hand, racetrack-like geometries^13,58^ with stationary coils as presented in Figure 4a are possible, sensing moving edge dislocations, domain walls, and other mobile topological defects via their nonlinear response.

The results presented in this work clarify the interaction of topologically nontrivial spin textures and domain walls in chiral magnets with external magnetic fields, revealing a pronounced nonlinear response due to field-induced surfaces charges. The additional surface charges lead to a more long-ranged interaction compared to the unperturbed magnetic state, visible as so-called susceptibility contrast in MFM measurements. In general, these charges facilitate new opportunities for the detection and differentiation of nanoscale spin textures using, for example, nanoSQUIDs. On the basis of the nonlinear response, the detection sensitivity can be improved as demonstrated for two instructive examples, that is, a magnetic edge dislocation and a helimagnetic curvature wall. The proposed detection scheme is universal and, in principle, can be applied to all magnetic nano-objects that exhibit a different susceptibility than their surroundings, enabling sensing of otherwise hidden 1D and 2D spin textures. The latter is supported by the recent observation of enhanced magnetic susceptibility at antiferromagnetic domain walls in the topological insulator MnBi_2_Te_4_,^51^ expanding application opportunities into the realm of antiferromagnetic spintronics. Thus, in addition to the fundamental insight into the nanoscale physics of chiral magnets, this work introduces a viable read-out scheme for topological magnetic defects, and local spins arrangements in general, opening new possibilities for the design and fabrication of on-chip devices for spintronics.

## Supporting Information Available

Details about the sample preparation, magnetic force microscopy (MFM) measurements, micromagnetic simulations, additional data on the dependence of the MFM response on the tip lift height and separation of MFM signals. Note 1, methods; Note 2, experimental dependence of MFM signal on the tip lift height; Note 3, micromagnetic simulations of linear and nonlinear MFM response and their dependence on the tip lift height; Note 4, separation of the MFM response into magnetization and susceptibility contrast; Note 5, derivation of susceptibility contrast and magnetization contrast in MFM; Note 6: numerical calculation of the susceptibility (PDF)
