# Supplementary material for: Detection of Topological Spin Textures via Nonlinear Magnetic Responses
Source: Nano Lett. 2021 Dec 22;22(1):14–21. doi: 10.1021/acs.nanolett.1c02723 (PMC8759079; doi:10.1021/acs.nanolett.1c02723)
Supplement: Supplementary file 1 — nl1c02723_si_001.pdf [file nl1c02723_si_001.pdf]

Supporting information

Detection of Topological Spin Textures via Non-Linear Magnetic Responses

Mariia Stepanova[1,2#], Jan Masell[3,#], Erik Lysne[1,2,#], Peggy Schoenherr[4,5], Laura Köhler[6], Michael Paulsen[7], Alireza Qaiumzadeh[2], Naoya Kanazawa[8], Achim Rosch[9], Yoshinori Tokura[3,8,10], Arne Brataas[2], Markus Garst[6,11], and Dennis Meier[1,2,*]

[1]Department of Materials Science and Engineering, Norwegian University of Science and Technology (NTNU), Trondheim, 7491, Norway.

[2]Center for Quantum Spintronics, Department of Physics, Norwegian University of Science and Technology (NTNU), Trondheim, 7491, Norway.

[3]RIKEN Center for Emergent Matter Science (CEMS), Wako, 351-0198, Japan

[4]School of Materials Science and Engineering, UNSW Sydney, Sydney, NSW 2052, Australia

[5]ARC Centre of Excellence in Future Low-Energy Electronics Technologies (FLEET), UNSW Sydney, Sydney, NSW 2052, Australia

[6]Institute of Theoretical Solid State Physics, Karlsruhe Institute of Technology, 76049 Karlsruhe, Germany

[7]Physikalisch-Technische Bundesanstalt (PTB), Berlin, 10587, Germany

[8]Department of Applied Physics, University of Tokyo, Tokyo, 113-8656, Japan

[9]Institute for Theoretical Physics, University of Cologne, Cologne, 50937, Germany

[10]Tokyo College, University of Tokyo, Tokyo, 113–8656, Japan

[11]Institute for Quantum Materials and Technology, Karlsruhe Institute of Technology, 76021 Karlsruhe, Germany.

[#]The authors contributed equally to this work.

Note 1. Methods

Sample Preparation: Single crystals of FeGe were grown by the chemical vapor transport method. To achieve flat high-quality surfaces for MFM imaging, the samples were prepared by lapping and polishing to achieve a root mean square roughness of approximately 1 nm and cleaned with high-purity acetone and methanol, following the same procedure as described in Refs.[1,2].

Magnetic force microscopy: The MFM data was recorded using a commercial SPM system (NT-MDT NTEGRA Prima AFM). Magnetic probe tips (PPP-MFMR from Nanosensors) with force constant of 2.8 N/m and quality factor Q of about 200 were used. The tips possess a hard magnetic coating with effective magnetic moment of 10^{-16} A m^2 and have been magnetised by a permanent magnet prior to the measurements. Sample cooling was achieved using a water-cooled Peltier element and the measurements were carried out in N_2 atmosphere (with a few mBar overpressure) to prevent ice formation. The microscope was operated in two-pass MFM mode with the magnetic tip oscillating at its resonance frequency ($\approx$ 70 kHz) with an amplitude of $\approx$ 30 nm. During the first pass, a topography image was collected by scanning with the tip close to the sample surface. During the second pass, the tip was lifted 10-200 nm (in addition to 30 nm in the first pass) and retraced the measured topography to sense solely the magnetic interaction between the magnetic stray field of the sample and the magnetic tip. Between measurements with opposite magnetisation of the tip, the sample was heated to room temperature and the protective hood was removed to switch the tip magnetisation using a permanent magnet. Regions of interest were tracked using an optical microscope and the obtained MFM images were aligned using topographical features on the sample surface.

Simulations: We model the magnetization in FeGe in the presence of an MFM tip in an effective isotropic model. We assume that the magnetization of the MFM tip and hence also the stray field are constant. This model is similar to the micromagnetic model, but neglects contributions of the demagnetizing field within the sample as these significantly slow down our calculations while their corrections are assumed to be only marginal. The simulations were performed at $T = 0$ K using the following parameters for FeGe: $A = 8.78$ pJ m^{-1}, $D = 1.58$ mJ m^{-2}, and $M_s = 384$ kA m^{-1}.[3] For the numerics, we discretize the continuum theory on a regular mesh of cuboids (a_x, a_y, a_z) with $a_i \approx \lambda/16$ where $\lambda = 4\pi A/D$ is the wavelength of the helix. The rather coarse discretization is still suitable as we approximate derivatives by fourth order stencils.[4] The energy of the 3d setup is minimized by a (single precision) GPU-accelerated self-written software[5] where one boundary condition is von-Neumann (the surface to vacuum) and the opposite boundary is fixed to the bulk minimizer. The other boundaries are also fixed but were pre-relaxed under the constraints of one "bulk" and one "surface" edge. The thickness of the 3d slab is usually of the order $L_z \approx 2\lambda$ with additional tests for $L_z \approx 8\lambda$. For one pixel in a non-linear MFM-image, we relax the magnetization in a local spheroid for three different tip heights which we then use to compute the second derivative of the dipolar interaction energy.

Note 2. Experimental dependence of the MFM signal on the tip lift height

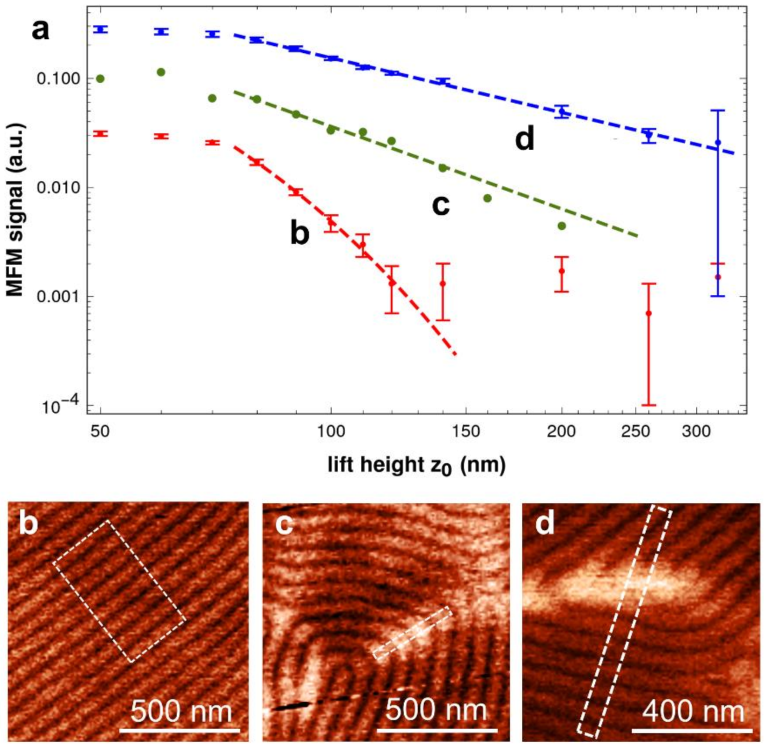

Figure S.1. Relation between MFM signal and lift height of the MFM tip. Red data points represent the evolution of the helix amplitude, evaluated based on an MFM images series recorded with varying lift height of the probe tip. Data points are derived by fitting a sinusoidal function to averaged data taken from the region marked by the dashed box in (1). The red dashed line is a fit with an exponential decay $I = I_0\, e^{-\frac{2\pi}{L} z_0}$ with $L = 100 \pm 10$ nm. Green data points show the evolution of the response at the zigzag domain wall (type II) in (2). Plotted is the peak value of the MFM phase signal, calculated as the difference between an averaged profile from the dashed box in (2) and the averaged signal from the adjacent helimagnetic background. The green dashed line is a polynomial fit $I \propto z_0^{-\alpha}$ with $\alpha = 2.5 \pm 0.5$. The blue data points are the peak value measured at the type III domain wall in (3), estimated by fitting a Gaussian function to the profile averaged over the dashed box in (3). The blue dashed line is a polynomial fit $I \propto z_0^{-\alpha}$ with $\alpha = 1.66 \pm 0.33$.

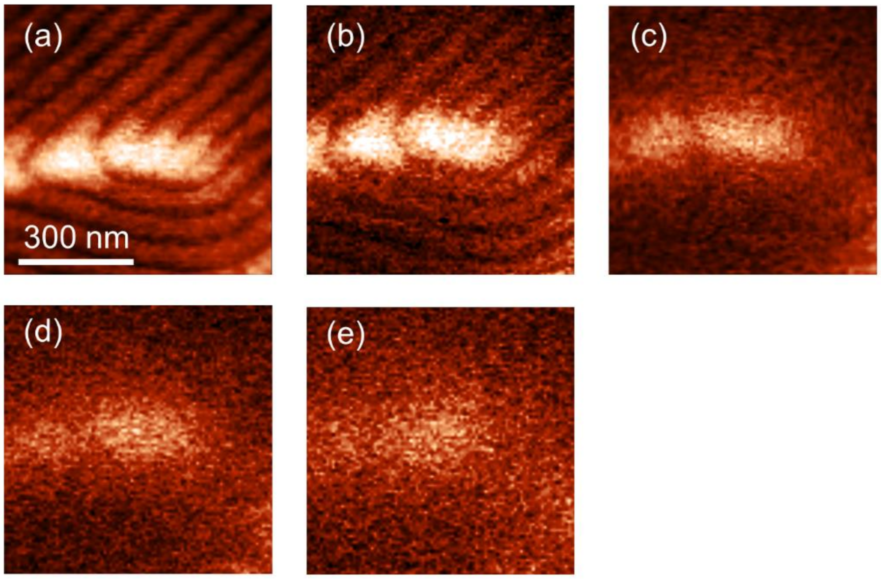

Figure S.2. MFM scans recorded at a type III domain wall with increasing lift height. (a) 60 nm, (b) 80 nm, (c) 100 nm, (d) 120 nm, (e) 160 nm. For smaller tip-sample distances (60 nm and 80 nm) both magnetization and susceptibility contrast are resolved, whereas for larger distances ($\gtrsim$ 100 nm) only the susceptibility contrast is detected.

Note 3. Micromagnetic simulations of linear and non-linear MFM response and their dependence on the tip lift height

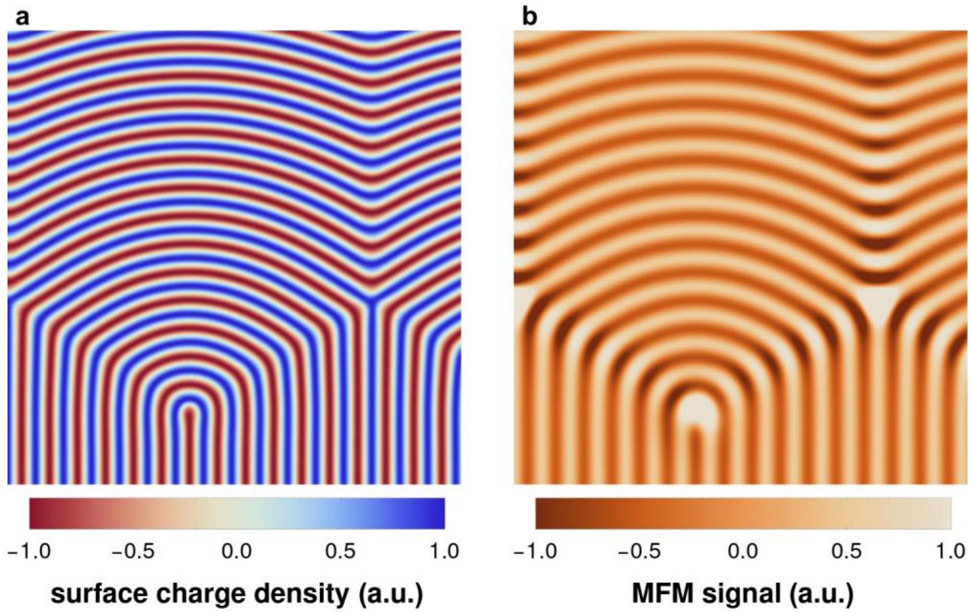

Figure S.3. Linear MFM signal in simulations. (a) Distribution of magnetic surface charges, i.e., out-of-plane components of the magnetization, of a zigzag domain wall at the surface of a bulk crystal. (b) Simulated linear MFM signal (lift height: 100 nm, tip moment: $2{\cdot}10^{-16}$ A m^2, same as in Figure 3b in the main text). The signal is enhanced at bent helices, both in the positive and negative direction. The color code is chosen such that the signal of the unperturbed helix oscillates between $\pm\frac{2}{3}$.

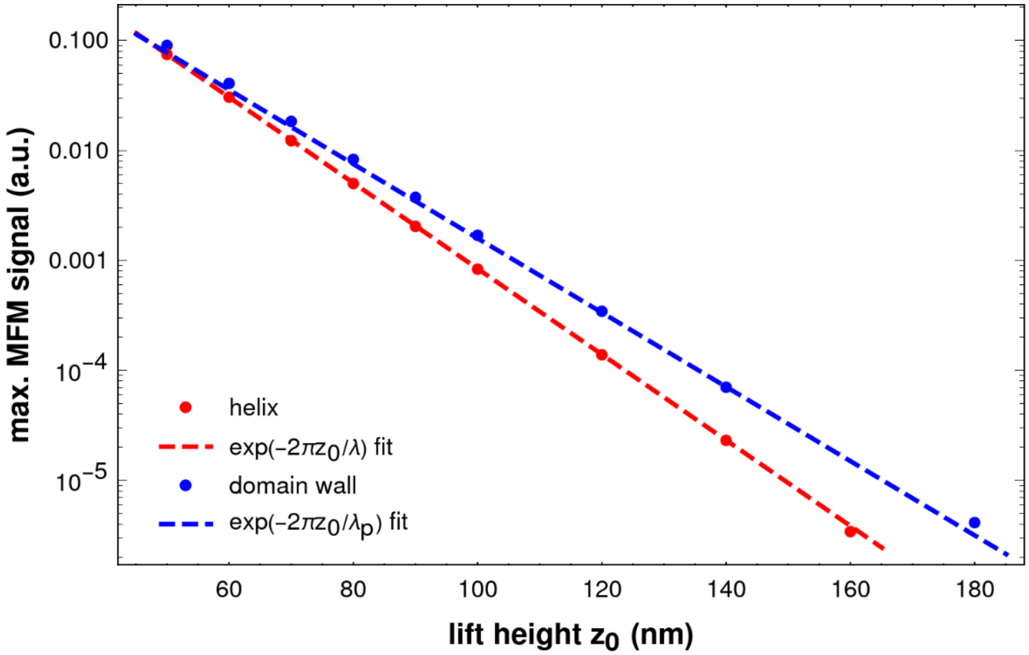

Figure S.4. Distance-dependence of simulated linear MFM signals. Red dots show the maximal signal on a helix without defects. Blue dots show the maximal signal on a curvature domain wall with an angle φ = 30° between the wall and the helical **q**-vector on either side. The tip-sample interaction is neglected. The data is well described by an exponential decay $I \propto e^{-\frac{2\pi}{\lambda}z_0}$ where z_0 is the lift height and λ is the local wavelength, i.e., λ = 70 nm for a helix without defects and $\lambda = \lambda_p$ 70 nm / cos(φ) for the curvature wall, see dashed lines.

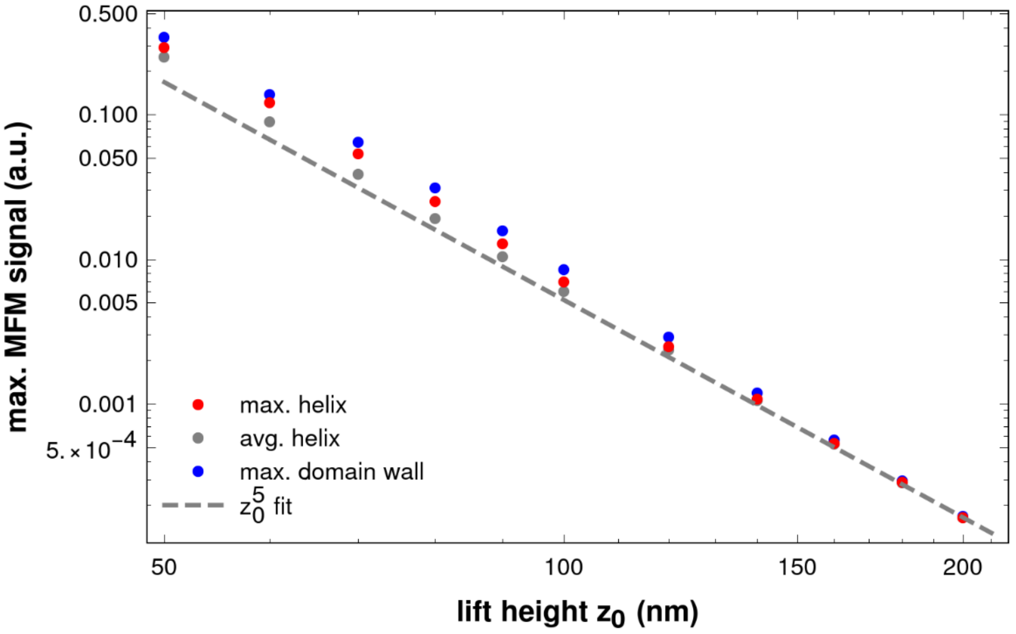

Figure S.5. Distance-dependence of simulated non-linear MFM signals. Red dots show the maximal signal on a helix without defects. Gray dots show the average signal on the same helix. Blue dots show the maximal signal on a curvature domain wall with an angle φ = 30° between the wall and the helical **q**-vector on either side. The tip is modelled as a dipole with moment 10^{-16} A m^2. In contrast to Figure S.4, the average signals do not vanish. Instead, the average signal on the helix is well described by a power law $I \propto z_0^5$, see Supporting Note 5 for details. Deviations from this trend are discussed in Figure S.6.

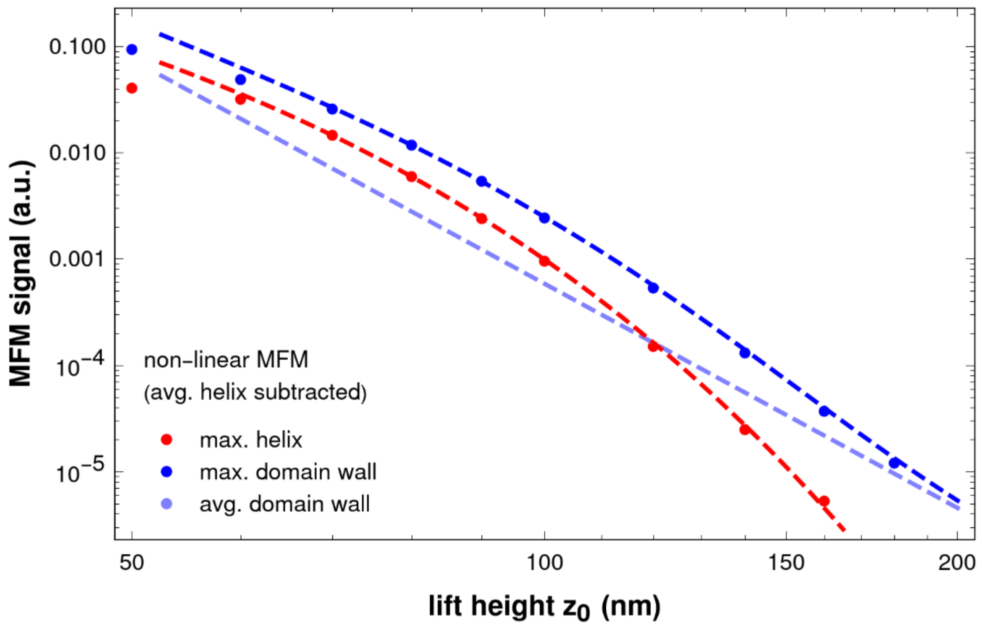

Figure S.6. Distance-dependence of simulated non-linear MFM signals. The average signal in the helical phase, see Figure S.5, has been subtracted. Red dots show the maximal signal on a helix without defects. Blue dots show the maximal signal on a curvature domain wall with an angle $\varphi = 30°$ between the wall and the helical $\boldsymbol{q}$-vector on either side. Light blue dots show the average signal on this domain wall. The tip is modelled as a dipole with moment 10^{-16} A m^2. The signal on the helix is well described by an exponential fit $I = I_0\, e^{-\frac{2\pi}{\lambda} z_0}$ where $\lambda = 70$ nm is the wavelength of the helix, see red dashed line, in agreement with the discussion of the magnetic signal in the Supporting Note 5, see equation (S.7). The average signal on the domain wall is well fitted by a power law $I \propto z_0^7$ which describes the susceptibility contrast. The discrepancy to the analytical prediction in the Supporting Note 5, see equation (S.11), probably arises because we simulate a slab of finite thickness and not an infinitely thick specimen. Finally, the maximal signals on the domain wall are well fitted by the power law for the susceptibility contrast plus an additional exponential decay $\propto e^{-\frac{2\pi}{\lambda/\cos(\phi)} z_0}$ for the magnetic signal, see Supporting Note 5 for details.

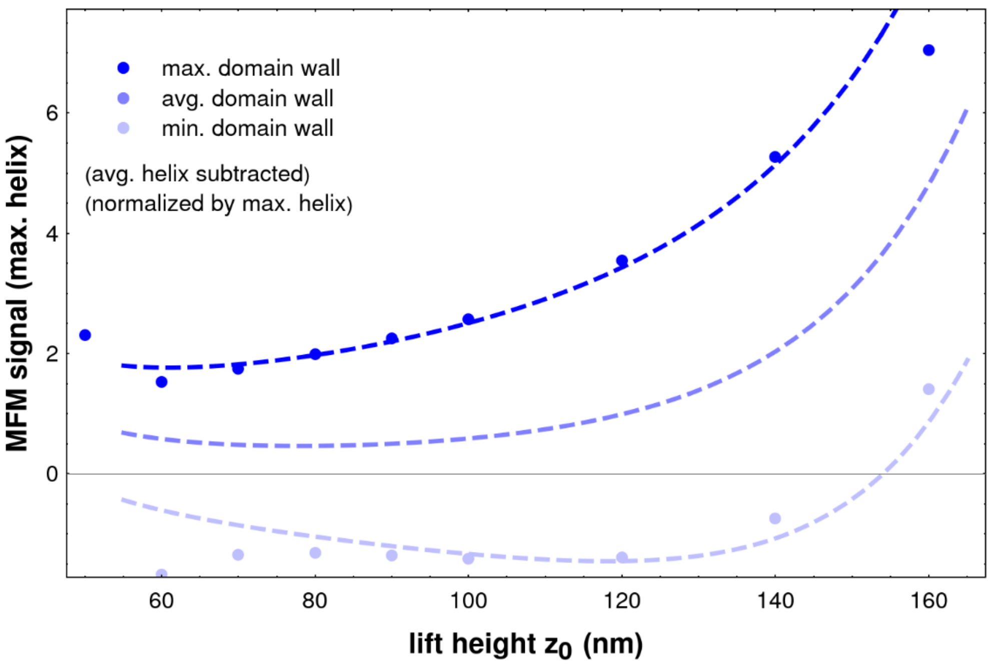

Figure S.7. Distance-dependence of simulated non-linear MFM signals. The average signal in the helical phase, see Figure S.5, has been subtracted. Data points show the maxima/average/minima of the MFM signal on a curvature domain wall (φ = 30°) normalized by the maximal signal on the helical phase without defects. The tip is modelled as a dipole with moment 10^{-16} A m^2. The dashed lines are the same fits as in Figure S.6 (for the minima we just inverted the sign of the extra exponential decay on the domain wall). The graph illustrates how the signal on the domain wall becomes increasingly brighter than the signal on the helix as the distance between the tip and the sample is increased.

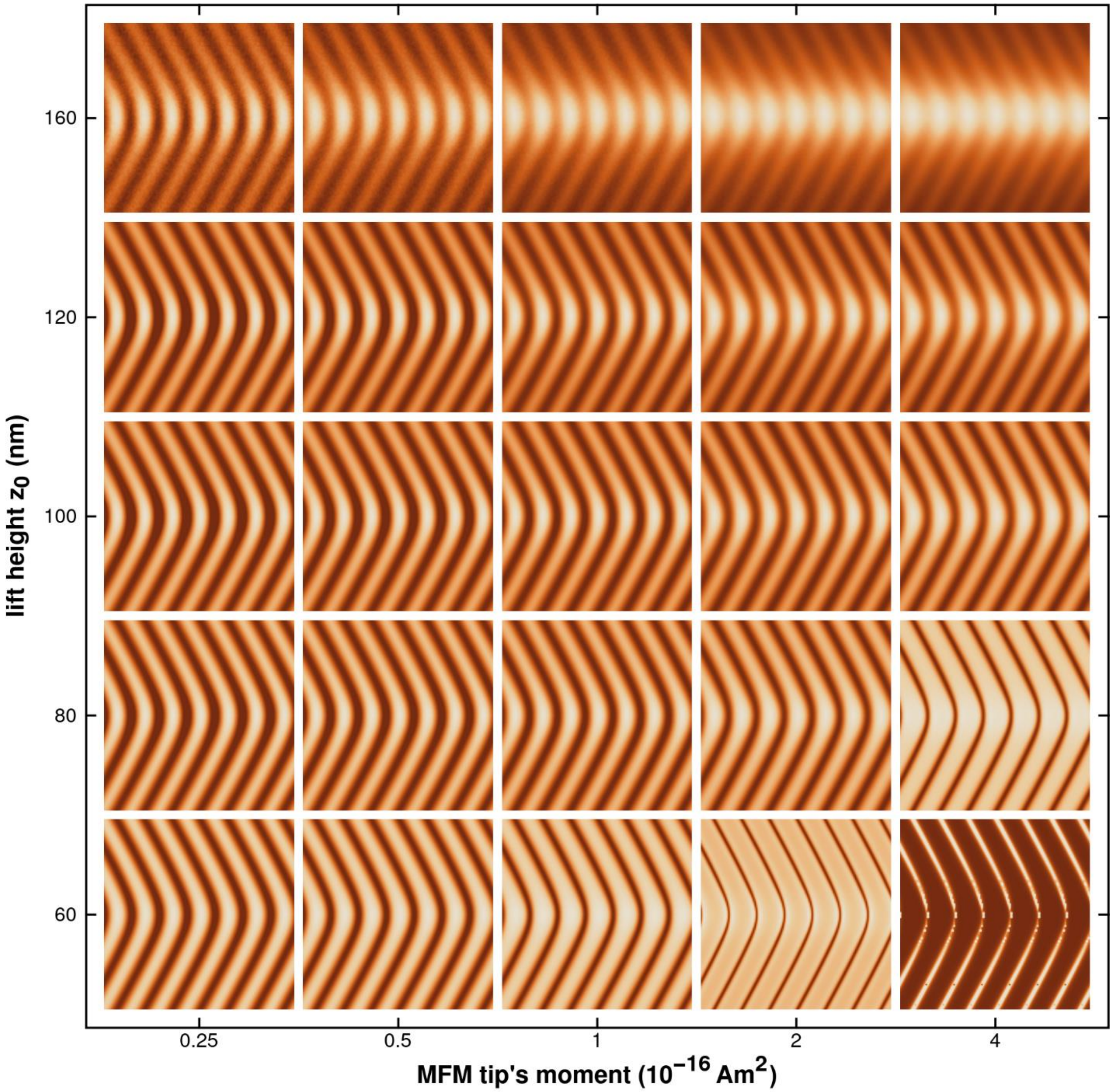

Figure S.8. Tip-dependence of simulated non-linear MFM signals. The panel matrix shows MFM maps for various lift heights (rows) and tip magnetizations (columns), simulated for a curvature domain wall with an angle $\varphi = 30°$ between the wall and the helical $\boldsymbol{q}$-vector on either side. Lift heights and tip moments are indicated in the figure.

Note 4. Separation of the MFM response into magnetization and susceptibility contrast.

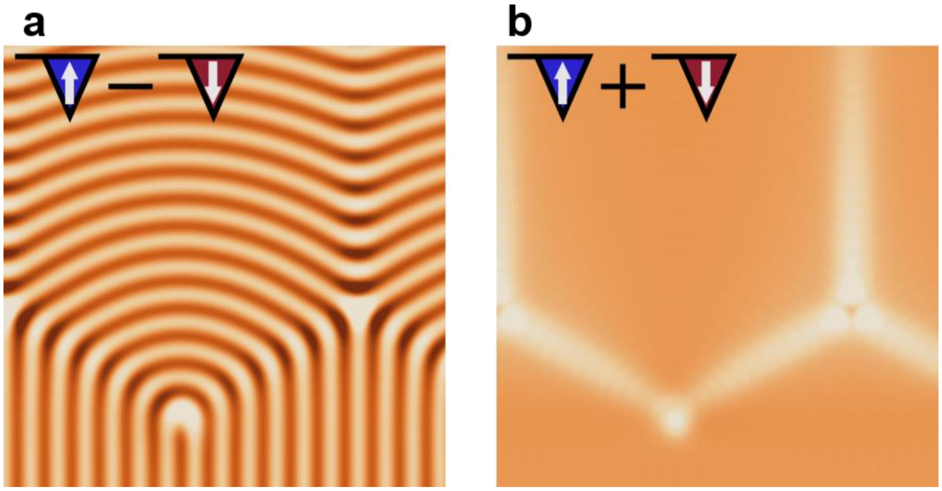

Figure S.9. Separation of the non-linear MFM signal in simulations into magnetization and susceptibility contrast. (a) magnetization contrast, and (b) susceptibility contrast, based on Figure 3b in the main text. The magnetization contrast is defined as $I_m = (I_{up} - I_{down})/2$ and the susceptibility contrast as $I_\chi = (I_{up} + I_{down})/2$, where I_{down} and I_{up} are the contrasts for a "down"-polarized and "up"-polarized tip, c.f. Figure 3b and d in the main text, respectively. See Supporting Note 5 for details.

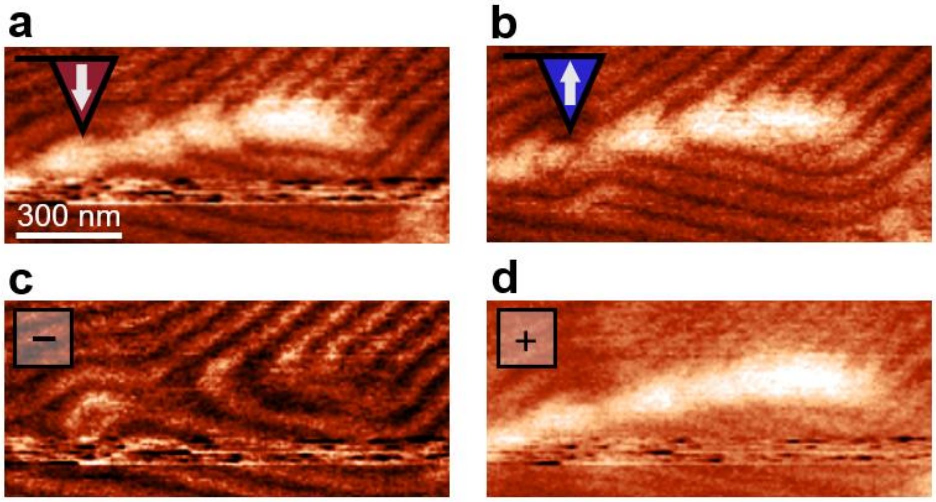

Figure S.10. Experimental separation of magnetization and susceptibility contrast. (a) and (b) MFM images of the same region measured with the tip magnetized “down” and “up”, respectively. The area shown here corresponds to the region marked by the white dashed line in the larger MFM scan presented in Figure S.11. (c) Magnetization contrast image gained by taking the difference of the data presented in (a) and (b), corresponding to I_m. (d) Sum of the MFM data in (a) and (b), showing the susceptibility contrast image (I_χ).

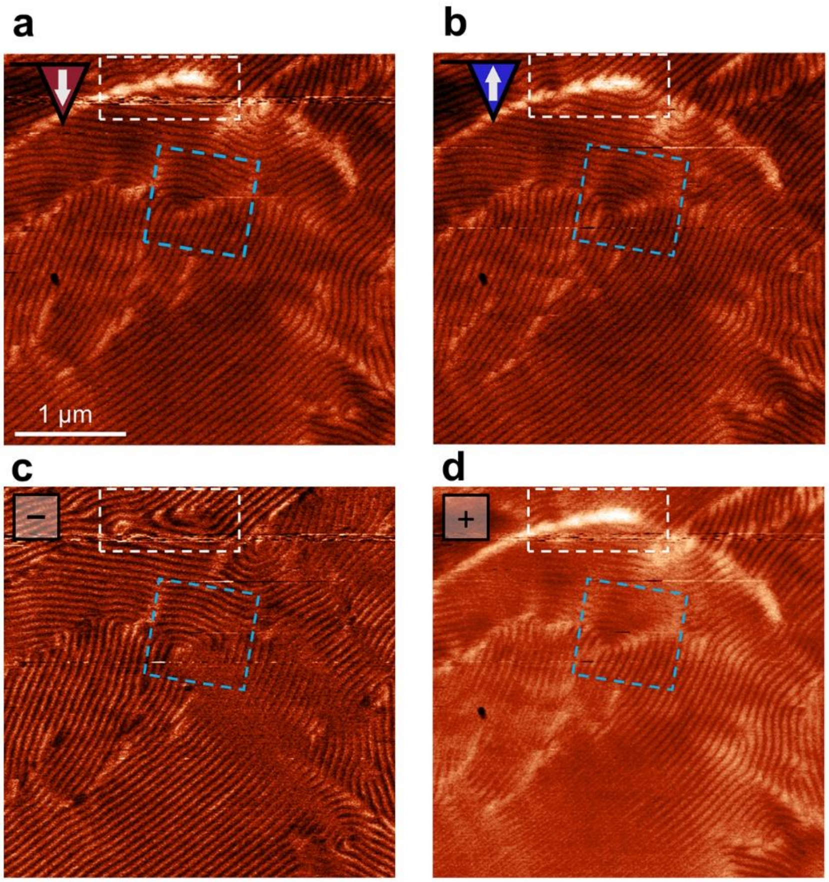

Figure. S.11. Overview MFM images taken with oppositely magnetized tip. (a) and (b) MFM images of the same region (3.8 µm × 3.8 µm) measured with the tip magnetized "down" and "up", respectively. To change the magnetization of the tip, the sample was transiently heated to room temperature, i.e., $T > T_N$. The region discussed in Figure 3 in the main text is marked by the blue dashed line and the area presented in Figure S.10 is marked by the white dashed line. (c) Magnetization contrast image gained by taking the difference of the data presented in (a) and (b), corresponding to I_m. (d), Sum of the MFM data in (a) and (b), showing the susceptibility contrast I_χ. In specific regions where the local spin texture is restored almost completely after heating above T_N (most likely due to structural defects that act as pinning sites), a clear separation into magnetization and susceptibility contributions is possible (see white dashed box in (c) and (d)). In general, however, the spin textures observed before and after T_N are not identical, leading to

blurring as MFM images are added or subtracted. Despite this effect, the experiments reveal that helix-related MFM contrasts invert along with the tip magnetization as explained in detail in the main text, considering the region marked by the blue dashed box.

Note 5. Derivation of susceptibility contrast and magnetization contrast in MFM

Formulation of the non-linear problem. When computing the energy of a magnetic tip, one has to consider that the presence of the tip leads to a distortion of the magnetization of the sample. If this distortion is small, it can be calculated from the susceptibility $\chi(\boldsymbol{r},\boldsymbol{r}')$ of the sample and the dipolar field $\int d\boldsymbol{r}'\, \chi_d^{-1}(\boldsymbol{r}-\boldsymbol{r}')\boldsymbol{M}_{tip}(\boldsymbol{r}')$ due to the magnetization $\boldsymbol{M}_{tip}(\boldsymbol{r}')$ of the tip with

$$\left(\chi_d^{-1}(\boldsymbol{r})\right)_{ij} = \frac{\mu_0}{4\pi}\frac{3\, r_i r_j - \delta_{ij} r^2}{r^5} = \frac{\mu_0}{4\pi}\partial_{r_i}\partial_{r_j}\frac{1}{r}. \tag{S.1}$$

The total change of magnetic energy due to the presence of the tip can then be approximated as

$$U \approx U_m + U_\chi \tag{S.2}$$

where the two contributions are sensitive to the magnetization $\boldsymbol{M}$ and susceptibility χ of the sample, respectively, with

$$U_m = -\int d\boldsymbol{r} d\boldsymbol{r}' \boldsymbol{M}_{tip}(\boldsymbol{r})\chi_d^{-1}(\boldsymbol{r}-\boldsymbol{r}')\boldsymbol{M}(\boldsymbol{r}') \tag{S.3}$$

$$U_\chi = -\frac{1}{2}\int d\boldsymbol{r} d\boldsymbol{r}' d\boldsymbol{r}_1 d\boldsymbol{r}_2 \boldsymbol{M}_{tip}(\boldsymbol{r})\chi_d^{-1}(\boldsymbol{r}-\boldsymbol{r}_1)\,\chi(\boldsymbol{r}_1,\boldsymbol{r}_2)\,\chi_d^{-1}(\boldsymbol{r}_2-\boldsymbol{r}')\boldsymbol{M}_{tip}(\boldsymbol{r}')\,. \tag{S.4}$$

As MFM measures changes in the oscillation frequency of the tip, it is proportional to $-\partial_z^2 U$. Thus, we can define both the magnetization signal and susceptibility signal

$$I_m = -\partial_z^2 U_m\,, \quad I_\chi = -\partial_z^2 U_\chi\,. \tag{S.5}$$

By subtracting and adding the MFM signal for a reversed tip, one can measure these two contributions separately, c.f. Figures S.9-11.

The relative strength of I_m and I_χ depends on the magnetization of the MFM tip, $\mathbf{M}_{\text{tip}}(\mathbf{r})$. In our case the tip is of pyramidal shape and covered by a thin magnetic layer. Unfortunately, the domain

structure of the magnetization is not known. We will therefore discuss below two models for the tip magnetization, called “dipolar tip” and “pyramidal tip”.

For the dipolar tip, we simply approximate the tip by a point-like dipole at position $\boldsymbol{r}_0$ as $\boldsymbol{M}_{tip}(\boldsymbol{r}) = \boldsymbol{m}_{tip}\delta^3(\boldsymbol{r} - \boldsymbol{r}_0)$. For the pyramidal tip, in contrast, we assume that the sides of a pyramidal tip are covered with a thin magnetic layer of uniform width with a magnetization oriented in the z direction. We assume that both the thickness of the magnetic layer and the rounding of the tip of the pyramid are much smaller than the distance z_0 of the tip from the surface and that the height of the pyramid is much larger than the lift height z_0. Both conditions are met in our experiment. This allows to ignore the rounding and to approximate the tip as an infinitely long pyramid. The precise value of the opening angle of the pyramidal tip and the number of sides of the pyramid are not important for the following qualitative discussion which focusses on the qualitative dependence of I_m and I_χ on the distance z_0 of the tip from the surface. Technically, all integrals in equations (S.3) and (S.4) are evaluated using scaling arguments. In each case we checked numerically the validity of the scaling argument and the convergence of the integrals.

Magnetic signal. We consider a situation where the magnetization oscillates (approximately) periodically in one direction (we use the x-direction below) with period λ. In the helical phase, λ is simply the wavelength of the helix but along a curvature domain wall we have a larger (projected) wavelength $\lambda_p = \lambda/\cos(\phi)$. We study the case $2\pi z_0 > \lambda$ which is of relevance for both the helical phase and curvature domain walls. For the following qualitative analysis, it is useful to consider the Fourier transform of χ_d^{-1} parallel to the surface of the sample, which decays exponentially with z_0

$$\chi_d^{-1}(q_{||}, z_0) = \int dr_{||}\, e^{-iq_{||}r_{||}} \chi_d^{-1} \sim e^{-|q_{||}|z_0}\,. \tag{S.6}$$

In the helical phase and at curvature domain walls, the average magnetization vanishes. Therefore, the magnetization only has Fourier components which are multiples of $2\pi/\lambda$ (or $2\pi/\lambda_p$) and thus the oscillating magnetization signal is exponentially suppressed

$$I_m^{osc} \propto \cos\left(\frac{2\pi}{\lambda} x_0\right) e^{-\frac{2\pi}{\lambda} z_0} \tag{S.7}$$

both for the pyramidal tip and the dipolar tip. At a curvature domain wall, λ has to be replaced with $\lambda_p > \lambda$, resulting in a slower decay of the signal with the lift height z_0, see Figure S.3.

Close to more complex textures, in contrast, there can be a net average magnetization along the domain wall and, hence, the $q_{||} = 0$ Fourier components contribute. In this case we obtain

$$I_m \propto \begin{cases} \frac{1}{z_0^3}, & \text{dipolar tip} \\ \frac{1}{z_0}, & \text{pyramidal tip} \end{cases}\,. \tag{S.8}$$

The two-dimensional integral over the surfaces of the pyramid is responsible for the slower decay of its signal by two powers of z_0. For a rod-like defect oriented perpendicular to the surface (i.e., realizing a point-like defect on the surface), the signal decays with one power of z_0 higher

$$I_m \propto \begin{cases} \frac{1}{z_0^4}, & \text{dipolar tip} \\ \frac{1}{z_0^2}, & \text{pyramidal tip} \end{cases}\,. \tag{S.9}$$

Here we assumed that z_0 is larger than the width of the defect.

Susceptibility signal. The susceptibility χ can also be split into a constant part I_χ and an oscillating part I_χ^{osc}. In the helical phase without defects, by power-counting, we find that the non-oscillating part of the susceptibility signal yields a contribution

$$I_\chi \propto \begin{cases} \dfrac{1}{z_0^5}, & \text{dipolar tip} \\ \dfrac{1}{z_0}, & \text{pyramidal tip} \end{cases} . \qquad \text{(S.10)}$$

Note that the two signals differ by four powers of z_0 as one has to integrate in equation (S.4) twice over the two-dimensional surface of the MFM tip.

The difference $\Delta I_\chi = I_\chi^{dw} - I_\chi^h$ of the signal on top of the domain wall, I_χ^{dw}, and in the helical phase, I_χ^h, decays with one power of z_0 faster (assuming again that z_0 is larger than the width of the domain wall)

$$\Delta I_\chi \propto \begin{cases} \dfrac{1}{z_0^6}, & \text{dipolar tip} \\ \dfrac{1}{z_0^2}, & \text{pyramidal tip} \end{cases} . \qquad \text{(S.11)}$$

By analyzing equation (S.8), one can show that the oscillating part of I_χ which arises from the oscillating part of the susceptibility decays exponentially

$$I_\chi^{osc} \propto \cos\left(\frac{2\pi}{\lambda} x\right) p(z_0)\, e^{-\frac{2\pi}{\lambda} z_0}, \qquad \text{(S.12)}$$

similar to I_m^{osc} but with a much smaller (z_0-dependent) prefactor $p(z_0)$.

Conclusions. Our analytical analysis qualitatively explains our main experimental and numerical observations. The experiment operates in a regime where the exponentially suppressed oscillatory magnetization signal I_m^{osc} is of similar size compared to the susceptibility signal,

ΔI_χ. For larger distances z_0 between the tip and the sample, the susceptibility signal dominates close to curvature domain walls which do not have an average magnetization. The exponentially suppressed oscillating contrast from the susceptibility signal, I_χ^{osc}, is never observable in our experiments and barely visible in the numerics (see Figure S.9). Non-periodic features or features with a period much larger than z_0 can, however, easily be resolved, see, e.g., Figure S.9.

In Figure S.1 the z_0 dependence of the MFM signal is shown in three cases. First, on top of the helical background, we observe an exponential decay of the oscillatory part of the signal, see equation (S.7). The fitted decay length is nominally about 50% larger than predicted, probably due to the limited fitting range and the relatively small values of z_0 available for the fit. In regions 2 and 3 of Figure S.1 a much slower decay of the signal is observed. In region 2, where the magnetization can locally be described by a curvature domain wall, the signal arises most likely from the susceptibility. The fitted decay $\propto \frac{1}{z_0^\alpha}$ with $\alpha \approx 2.5 \pm 0.5$ appears to be slightly faster than the expected $\frac{1}{z_0^2}$ for an idealized pyramidal tip, equation (S.11). The discrepancy can either arise due to the rather broad and more complex magnetic structure of the sample or from possible magnetic domains in our tip magnetization. Finally, in region 3, we do not expect a pure power law due to the rather complex magnetic structure which, most likely, is also characterized by a net magnetization. Therefore both I_χ and I_m may contribute to the signal. The fitted power law with exponent $\alpha \approx 1.66 \pm 0.33$ consistent with $\frac{1}{z_0^2}$ predicted both for the magnetization signal of a rod-like defect, equation (S.9), and for the susceptibility contrast of a domain wall, equation (S.11).

Note 6. Numerical calculation of the susceptibility

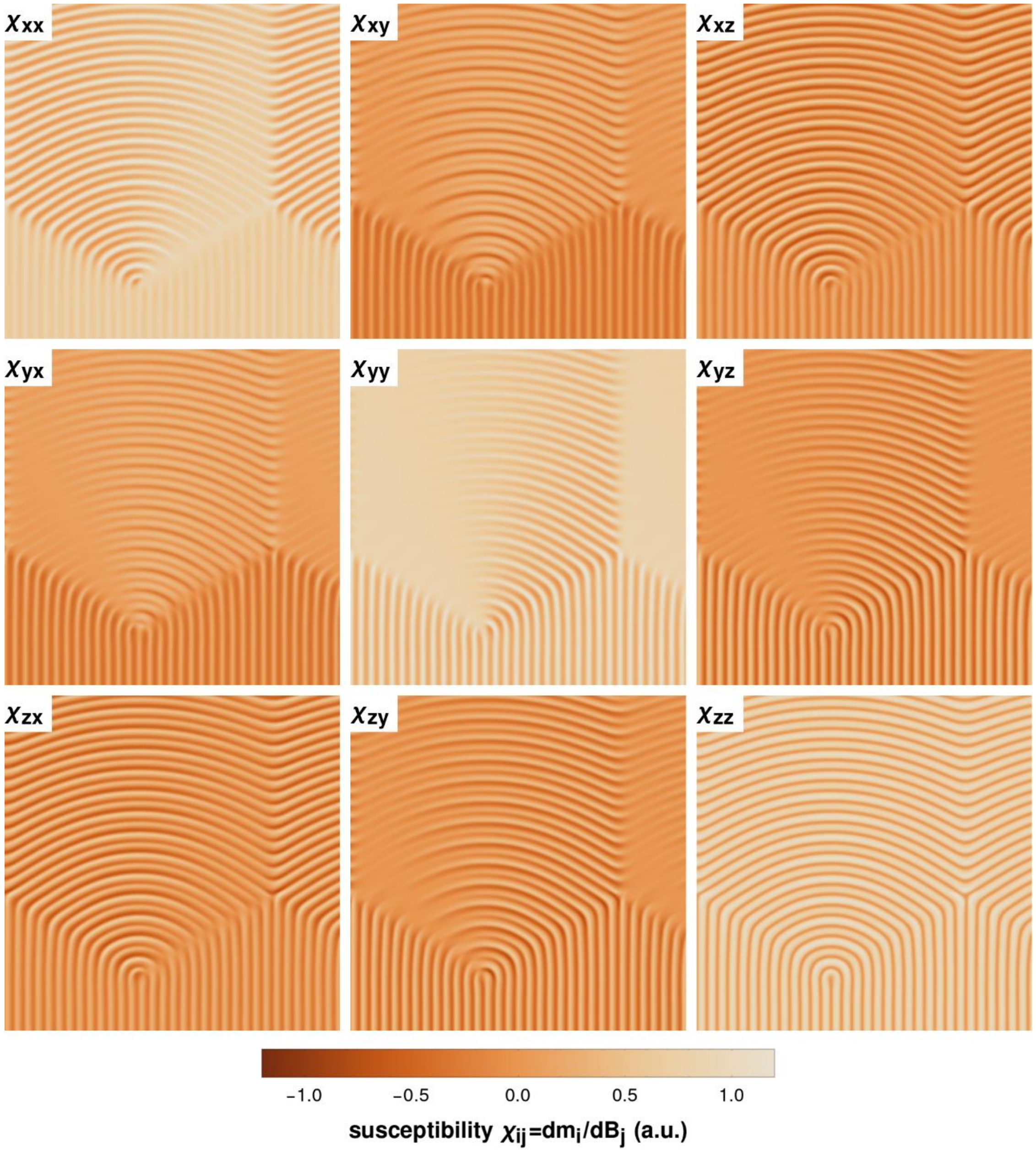

Figure S.12. Numerically calculated susceptibility $\chi_{ij}(r_1, r_2) = \frac{d\boldsymbol{m}_i(\boldsymbol{r}_1)}{d\boldsymbol{B}_j(\boldsymbol{r}_2)}$ in the domain wall shown in Figures S.3 and S.9. Rows indicate $i = x, y, z$ and columns $j = x, y, z$. Only results for $\boldsymbol{r}_1 = \boldsymbol{r}_2 = \boldsymbol{r}$ are shown, with $\boldsymbol{r}$ restricted to the top layer of the three-dimensional simulated magnetization.

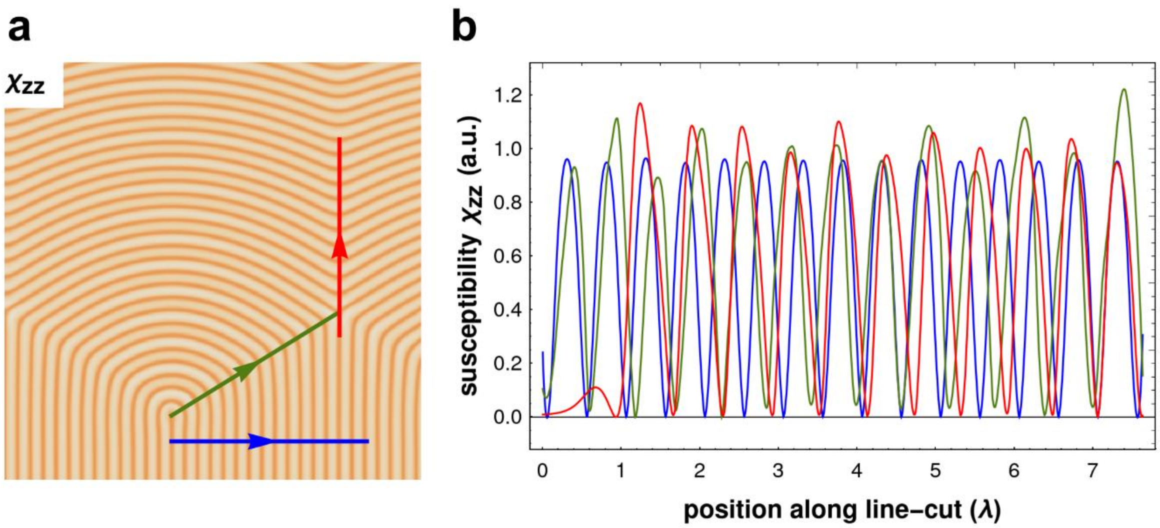

Figure S.13. Line scans of the susceptibility component $\chi_{zz}(\boldsymbol{r},\boldsymbol{r})$, see also Figure S.12. The paths in the two-dimensional plane are indicated in (a) with an arrow indicating the scanning direction. This panel corresponds to the bottom right panel of Figure S12. (b) shows the data along the line scans in the corresponding color. It clearly visualizes what could hardly be extracted from Figure S.12, namely an enhancement of the susceptibility at the bent helix.

REFERENCES

(1) Dussaux, A.; Schoenherr, P.; Koumpouras, K.; Chico, J.; Chang, K.; Lorenzelli, L.; Kanazawa, N.; Tokura, Y.; Garst, M.; Bergman, A.; Degen, C. L.; Meier, D. Local Dynamics of Topological Magnetic Defects in the Itinerant Helimagnet FeGe. *Nat Commun* **2016**, *7* (1), 12430. https://doi.org/10.1038/ncomms12430.

(2) Schoenherr, P.; Müller, J.; Köhler, L.; Rosch, A.; Kanazawa, N.; Tokura, Y.; Garst, M.; Meier, D. Topological Domain Walls in Helimagnets. *Nature Phys* **2018**, *14* (5), 465–468. https://doi.org/10.1038/s41567-018-0056-5.

(3) Beg, M.; Carey, R.; Wang, W.; Cortés-Ortuño, D.; Vousden, M.; Bisotti, M.-A.; Albert, M.; Chernyshenko, D.; Hovorka, O.; Stamps, R. L.; Fangohr, H. Ground State Search, Hysteretic Behaviour and Reversal Mechanism of Skyrmionic Textures in Confined Helimagnetic Nanostructures. *Scientific Reports* **2015**, *5* (1), 17137. https://doi.org/10.1038/srep17137.

(4) Miltat, J. E.; Donahue, M. J. Numerical Micromagnetics: Finite Difference Methods. In *Handbook of Magnetism and Advanced Magnetic Materials*; American Cancer Society, 2007.

(5) Masell, J.; Yu, X.; Kanazawa, N.; Tokura, Y.; Nagaosa, N. Combing the Helical Phase of Chiral Magnets with Electric Currents. *Phys. Rev. B* **2020**, *102* (18), 180402. https://doi.org/10.1103/PhysRevB.102.180402.
